# A Mesozoic bird from Gondwana preserving feathers

**DOI:** 10.1038/ncomms8141

**Published:** 2015-06-02

**Authors:** Ismar de Souza Carvalho, Fernando E. Novas, Federico L. Agnolín, Marcelo P. Isasi, Francisco I. Freitas, José A. Andrade

**Affiliations:** 1 Universidade Federal do Rio de Janeiro, Departamento de Geologia, Avenida Athos da Silveira Ramos, 274, Rio de Janeiro CCMN/IGEO 21.949-900, Brazil; 2 CONICET, Museo Argentino de Ciencias Naturales ‘Bernardino Rivadavia', Avenida Angel Gallardo 470, 1405 Buenos Aires, Argentina; 3 Fundación de Historia Natural ‘Félix de Azara', Universidad Maimónides, Hidalgo 775, C1405BDB Buenos Aires, Argentina; 4 Geopark Araripe, Rua Carolino Sucupira s/n°, Pimenta, 105 Centro, 63.100-490 Ceará, Brazil; 5 Departamento Nacional da Produção Mineral, Ceará, Praça da Sé, 105 Centro, 63.100-440 Ceará, Brazil

## Abstract

The fossil record of birds in the Mesozoic of Gondwana is mostly based on isolated and often poorly preserved specimens, none of which has preserved details on feather anatomy. We provide the description of a fossil bird represented by a skeleton with feathers from the Early Cretaceous of Gondwana (NE Brazil). The specimen sheds light on the homology and 3D structure of the rachis-dominated feathers, previously known from two-dimensional slabs. The rectrices exhibit a row of rounded spots, probably corresponding to some original colour pattern. The specimen supports the identification of the feather scapus as the rachis, which is notably robust and elliptical in cross-section. In spite of its juvenile nature, the tail plumage resembles the feathering of adult individuals of modern birds. Documentation of rachis-dominated tail in South American enantiornithines broadens the paleobiogeographic distribution of basal birds with this tail feather morphotype, up to now only reported from China.

## Abstract (web summary)

Fossils of Cretaceous birds with feathers are rare and known mostly from China. Here, the authors show an enantiornithine bird from the Lower Cretaceous of Brazil with a fully articulated skeleton and rachis-dominated tail feathers, which has implications for our understanding of feather evolution.

## Graphical abstract

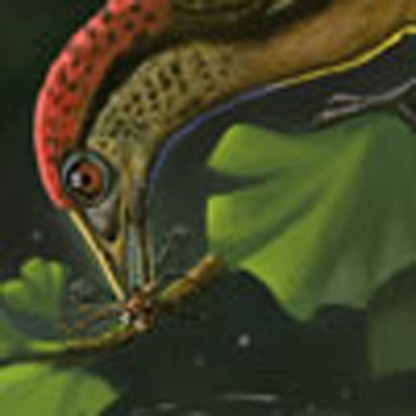

Cretaceous birds with feathers are very rare fossils with exceptional preservation. Most specimens and valuable information on feathers in early birds have been recovered from the Lower Cretaceous Jehol Group (Hauterivian through Aptian) in NE China (for example, see refs 1, 2). The Jehol fossil record comprises an extraordinary taxonomic diversity of basal birds preserving feathers including *Jeholornis*, Confuciusornithiformes and Enantiornithes, among others^2^,^3^. This evidence currently constitutes the most informative source to understand the early evolution of bird feathers. However, some skeletal remains associated with poorly preserved feathers have also been recovered in the Dundargalant Gorizont (Hauterivian-Barremian) of Mongolia^4^, and the Formación Calizas de La Huérguina (Barremian) beds from Spain (for example, see ref. 5).

Among the most curious fossil feather morphotypes is the ribbon-like or rachis-dominated type of feather (see refs 2, 6). They are usually described as proximally ribbon-like with distally restricted barbs, a morphology not documented among living birds^7^. Because most fossils are preserved in two dimensions, the detailed anatomy of these tail feathers still remains in debate. They have been variously suggested as representing an scale-like structure intermediate in morphology between the reptilian and bird integuments, a modified pennaceous feather, or a unique type of primitive feather^2^,^3^,^8^,^9^,^10^,^11^,^12^.

Here we present the discovery of a fully articulated skeleton associated with feathers, belonging to a minute enantiornithine bird from the Crato Formation (Lower Cretaceous) of Brazil. The specimen constitutes the most complete avian specimen of Early Cretaceous age from Gondwana; more importantly, it sheds light on the anatomical structure and probable function of the peculiar rachis-dominated tail feathers. Notably, the new specimen preserves feathers in relief; thus helping to recognize key features of the rachis-dominated feathers.

## Results

### Systematic paleontology

Aves Linnaeus, 1758Ornithothoraces Chiappe, 1996Enantiornithes Walker, 1981Euenantiornithes Chiappe and Walker, 2002Indeterminate genus and species

### Referred material

UFRJ-DG (Universidade Federal do Rio de Janeiro, Department of Geology collection) 031 Av, partial skeleton of a possible juvenile specimen preserved in slab and counterslab (Supplementary Fig. 1), including poorly preserved skull bones, fore- and hindlimbs, portions of vertebral column, and most of both pectoral and pelvic girdles (see details in Supplementary Note 1). The skeleton is exposed in lateral view, but the proximal caudal vertebrae and pygostyle are exposed dorsally. The same applies to the tail feathers, attached to the pygostyle.

The very small body size (Supplementary Note 2), large orbit, elongate caudal series, poorly developed proximal humerus and distal ends of other long bones (femur, tibia), as well as the lack of fusion in the metatarsus indicates that the specimen is probably a juvenile^13^,^14^,^15^.

### Locality and horizon

Pedra Branca Mine, Nova Olinda County, Ceará State, Brazil (7° 6′51.9″S, 39° 41′46.9″W). Araripe Basin, Crato Formation (Early Cretaceous, Aptian). This formation has yielded abundant and exceptionally preserved fossils of a large variety of plants and animals, representing one of the best well-known terrestrial ecosystems for the Early Cretaceous^16^. Isolated feathers probably belonging to birds have been described from these beds^17^,^18^, as well as succinct reports on avian skeletons associated with poorly preserved feathers 19.

### Description and comparisons

**Figure 1:**
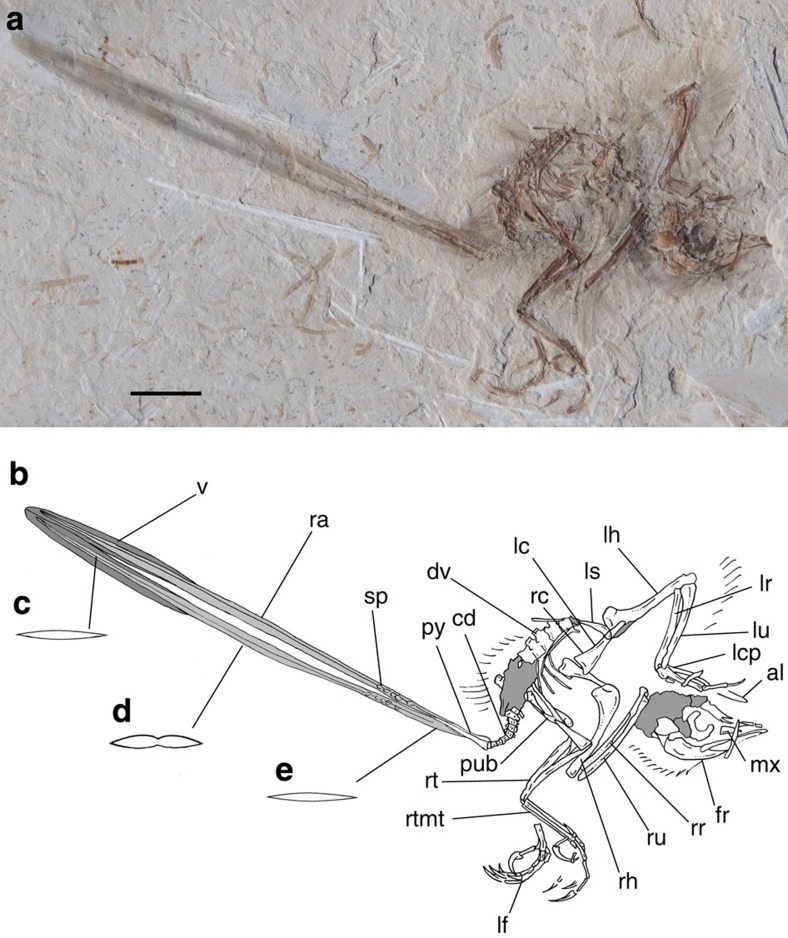
Main slab and interpretative drawing of specimen UFRJ-DG 031 Av. (**a**) Main slab. (**b**) Interpretative drawing of the skeleton and feathers. (**c**) Reconstructed cross-section at the level of distal vanes of the feathers. (**d**) Reconstructed cross-section at mid-length of the rachis. (**e**) Reconstructed cross-section of the calamus. al, alula; cd, free caudal vertebrae; dv, dorsal vertebrae; fr, frontals; lc, left coracoid; lcp, left carpometacarpus; lf, left foot; lh, left humerus; lr, left radius; ls, left scapula; lu, left ulna; mx, maxilla; pub, pubes; py, pygostyle; ra, rachis; rc, right coracoid; rh, right humerus; rr, right radius; rt, right tibiotarsus; rtmt, right metatarsals; ru, right ulna; sp, colour spots; v, vanes. Dark grey represents the vanes, light grey represents the scapus. Scale bar, 10 mm.

The specimen is the size of a hummingbird (approximately 6 cm from snout to tip of pygostile; Fig. 1; Supplementary Fig. 2). The skull is poorly preserved (Supplementary Note 1). The parietals and frontals are dorsally convex, indicating a vaulted braincase. The maxilla is subtriangular and the presence of minute alveoli supports that it was toothed. The vertebral column is represented by some cervical, dorsal, sacral and caudal vertebrae. Cervical centra are elongate, lacking pneumatic foramina. The neural spines are dorsoventrally tall and subrectangular in lateral view. Mid-dorsal centra are proportionally short, resembling more the proportions of basal enantiornithines (for example, *Iberomesornis*^20^), rather than the elongate condition of derived enantiornithines (for example, *Gobipteryx*^21^). Deep excavations are present on the lateral surface of the centra, and parapophyses are located high on the centrum, as usual among Enantiornithes^22^. Dorsal neural spines are subrectangular in contour (Supplementary Fig. 3). Caudal vertebrae are represented by eight free segments distally articulated with a pygostyle (Supplementary Fig. 4). Free caudals have short centra (Supplementary Fig. 5). The pygostyle is cone-shaped, composed by eight fused vertebrae, and longer than the combined length of the free caudals. The proximal end of the pygostyle is cone-shaped, and bears two subparallel longitudinal ridges as diagnostic of Enantiornithes^23^.

The coracoid is elongate and narrow, lacking a procoracoid. The scapula bears a prominent and tapered acromion. The humeral head is rounded head, the bicipital tubercle is poorly developed, and the capital groove and the transverse ligament groove are both absent. This simple proximal humeral morphology is reminiscent of juvenile enantiornithes^13^, as well as basal enantiornithines (for example, *Iberomesornis*, *Eocathayornis*^24^,^25^), and different from the more complex anatomy of adult-derived enantiornithines (for example, *Martinavis, Enantiornis, Gobipteryx, Halimornis*^21^,^26^,^27^,^28^). The distal end of the humerus is transversely expanded, although not to the degree seen in most Euenantiornithes^28^. The ulna is nearly as long as the humerus, a condition that contrasts with the much shorter proportions of several Euenantiornithes^27^,^28^. The radius exhibits a longitudinal groove diagnostic of Euenantiornithes^20^. The manus is subequal to ulnar length. Metacarpal II is shorter and than metacarpal III, a diagnostic condition of Euenantiornithes^28^ (Supplementary Fig. 5).

The pelvic girdle is fragmentary. Distally, the pubes exhibit a well-developed symphysis. The femur is nearly straight, and the femoral head is dorsally oriented. The tibiotarsus length is slightly shorter than the femur. The metatarsals are elongate and transversely narrow (metatarsal III subequal in length to tibiotarsus; Supplementary Fig. 6). The distal end of metatarsal I is caudally deflected, as diagnostic of Enantiornithes^20^. Metatarsal II is relatively robust but not wider than metatarsal III, a condition similar to other Enantiornithes^20^. Metatarsal IV is transversely compressed, particularly at its distal end, and is thinner than the remaining metatarsals, as characteristic of Enantiornithes^20^. Pedal digit III is narrow and extremely elongate, being much longer than the corresponding metatarsal, a condition shared with Bohaiornithidae^29^. Phalanx 1-I is elongate and robust, being the stoutest element of the foot. Pedal unguals are elongate and slightly curved, as occurs in Bohaiornithidae^29^. Digit I ungual is strongly curved, much more than the remaining unguals, a condition regarded as diagnostic of Enantiornithes^30^.

**Figure 2:**
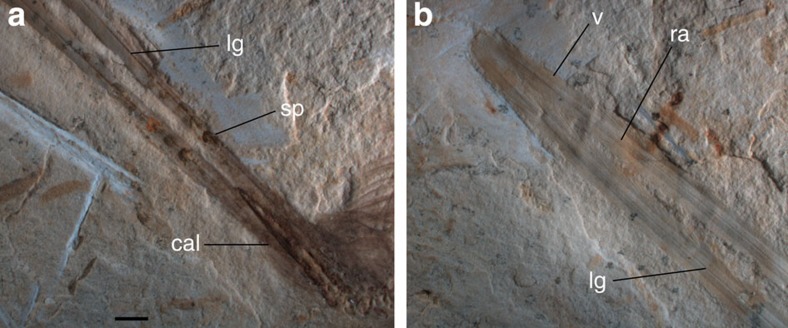
Details of tail feather of specimen UFRJ-DG 031 Av. (**a**) Proximal end. (**b**) Distal end. cal, calamus; lg, longitudinal groove; ra, rachis; sp, colour spot; v, vane. Scale bar, 2.5 mm.

The skeleton of UFRJ-DG 031 Av is covered by filamentous feathers, including approximately ten preserved secondary remiges anchored on the forearm. Left alula is represented by some asymmetrical feathers attached to digit I. Among preserved feathers, the paired rectrices are the most remarkable (Fig. 2). They are rachis dominated in morphology, similar to those already known among Enantiornithes and Confusiusornithiformes^2^,^5^,^6^. The rectrices are considerably elongate, being roughly 30% longer than length of skeleton. The femur/rectrix ratio is 0.16, similar to other enantiornithines (for example, *Dapingfangornis,* 0.15; *Paraprotopteryx*, 0.17 (ref. 2)). The rectrices insert on the third proximal pygostyle vertebra. The proximal portion of each feather that contacts the cone-shaped pygostyle is here interpreted as the calamus, whereas the remaining portion of feathers is identified as the rachis. The base of the rachis bears a row of five granulate spots, which we interpret as remnants of an ornamental colour pattern. The spots are distributed in a symmetrical paired line along both rectrices and shows comparable morphology, size, contour and colour. On this basis, we hypothesize that these spots may reflect the colour pattern of the feather and not a taphonomical artefact.

Each feather preserves a narrow groove extending from the base up to its distal end, traversing through the rows of spots. The rachis is slightly convex at mid-length, with the midline groove bisecting such a transverse convexity. The rachis flattens distally where it became vaned. The feather is symmetrical, as expressed by subequally sized vanes. As occurs in confuciusornithiforms and some enantiornithines^2^,^5^,^8^, the barbs are restricted to the distal 15% of feather length. Barbs size increase towards the distal end of feather. Each barb appears to be dorsoventrally thick and dorsally convex, and of uniform thickness and width for most of its length. No signs of the interlocking barbules are visible.

### Phylogenetic analysis

Phylogenetic relationships of UFRJ-DG 031 Av were analysed in the context of a comprehensive study of enantiornithines evolution (see Supplementary Notes 3 and 4; Supplementary Fig. 7). UFRJ-DG 031 Av exhibits the following synapomorphies of this clade of extinct birds^20^,^23^,^30^,^31^,^32^: pygostyle with ventrolateral processes, coracoid laterally convex, scapulocoracoid articulation with scapular pit and coracoidal tuber, metacarpal III more distally projected than metacarpal II, distal tarsals fused to proximal metatarsus, but remaining portion of metatarsals free and metatarsal I distal condyles caudally reflected (J-shaped). Derived features of Euenantiornithes present in UFRJ-DG 031 Av include radius with a posterior longitudinal groove, posterior femoral trochanter large and metatarsal IV significantly thinner than metatarsals II and III (ref. 28). Inclusion of UFRJ-DG 031 Av within data matrix offered by O'Connor and Zhou^31^ results in a polytomy of most enantiornithine genera. However, UFRJ-DG 031 Av exhibits some general similarities with *Iberomesornis*, *Pengornis, Eopengornis* and *Eoenantiornis*, such as a humeral head globose and projected further proximally, a capital groove poorly defined on proximal humerus, and dorsal vertebral centra craniocaudally short^7^,^20^,^32^,^33^. However, the differences noted in the diagnosis and description preclude considering UFRJ-DG 031 Av as nearly related to any of these taxa until more evidence becomes available.

## Discussion

A large number of Early Cretaceous basal pygostylians preserving feathers (that is, confuciusornithids, enantiornithines) show an enigmatic kind of tail formed by a pair of elongate rectrices known as ‘ribbon-like' or ‘rachis-dominated' feathers, which are unknown in living birds^1^,^2^,^5^,^6^. These feathers consist on a rachis-dominated proximal half with a barbed distal portion^8^. In contrast, *Sapeornis*, basal ornithuromorphs and a single possible enantiornithine, have a fan-shaped morphotype made up by a series of short rectrices, resembling that present in most living birds^2^,^34^.

Basic anatomy of rachis-dominated feathers remains difficult to interpret for two reasons: the absence of this kind of feather among living birds^2^ and the two-dimensional preservation of available specimens. This has lead to contradicting interpretations among authors. For example, Zhang *et al.*^6^,^35^ thought that the obscure longitudinal stripe represents a thin rachis, the lighter regions on each side of the ‘ribbon' constitutes undifferentiated vanes and the distal pennaceous portion forms a tapering extension of the ribbon-like rachis. Prum^35^, in contrast, hypothesized that the ribbon-like basal portion of the feather is formed by the dorsoventrally depressed and laterally expanded rachis. In the later context, some authors^2^,^8^,^12^,^36^ interpreted that the midline stripe of the scapus constitutes the longitudinal groove present on the ventral surface of rachis, and the flat regions of the sides of the stripe represent an expanded rachis.

In contrast with fossil bird specimens from China and Spain, the rectrices of UFRJ-DG 031 Av are preserved in relief, thus helping to confirm the midline dark stripe in the ribbon-like portion of the feather is not the rachis, but a longitudinal groove. Furthermore, the lateral expansions of the scapus are convex (and not flat, as feather vanes are) and accommodate barbs, thus this section may be interpreted as an expanded rachis. This evidence counters previous authors (for example, see ref. 3) interpreting that most of the shaft is the calamus, and that the rachis restricts to the distal pennaceous region. Foth^3^ proposed that the median longitudinal line represents the medullar cavity of calamus. However, in UFRJ-DG 031 Av, the midline stripe does not represent a medullar cavity, but a dorsal groove. Moreover, the presence of a string of spots (probably corresponding to colour patterns) constitutes additional evidence in support that this basal part of the feather was not embedded into the dermis, thus dismissing the interpretation that it corresponds to the calamus. We concur with Prum^37^ and O'Connor *et al.*^2^ in proposing the proximally narrow portion as an expanded rachis, rather than the calamus.

O'Connor *et al.*^2^ and Wang *et al.*^7^ interpreted that a narrow dark halo bordering the lateral margins of the rachis of rectricial feathers of Enantiornithes represented an undifferentiated vane. In sharp contrast with this pattern, specimen UFRJ-DG 031 Av shows that the base of the rachis is devoid of barbs, and that the first recognizable barbs emerge at nearly mid-length of the scapus and do not form an undifferentiated vane. Such differences may indicate that the rectricial morphology among enantiornithes was more variable than previously thought, and reveals a new aspect of variability not recognized before for the clade.

Information from UFRJ-DG 031 Av suggests that rachis-dominated tail feathers in Enantiornithes were very robust structures, dorsoventrally depressed, elliptical in cross-section and with a longitudinal groove running for most of its length on both dorsal and ventral surfaces. This morphology contrasts with feathers in extant birds in which the rachis is subquadrangular in cross-section and the groove is only on the ventral surface and bounded by two longitudinal ridges^15^.

Since the discovery of elongate rectricial feathers in *Confuciusornis*, authors mostly agree that such elongate structures may be sexually dimorphic and associated with sexual display, species recognition or visual communication^2^,^5^,^6^,^10^,^23^,^38^. The presence of a colour pattern on the tail base of UFRJ-DG 031 Av reinforces these interpretations. Because elongate rectrices were not present uniformly among basal pygostilians, even in members of a single species (for example, *Confuciusornis sanctus*^39^), it must be concluded that the absence or presence of these peculiar feathers was not decisive for body balance, thus countering interpretations favouring this view^40^. In living birds with elongate streamer feathers on the tail, the rectrices change their angle of attack and angle of spread (for example, see ref. 41), and are paired with an aerodynamic fan of normal length rectrices. Furthermore, the elongate outer rectrices of living birds become thinner past the point they are aerodynamic, where they are elongated past the other tail feathers^41^. However, ribbon-like feathers are sharply different from these tail feathers^11^,^12^: in confuciusornithiforms and enantiornithines, the rachis-dominated rectrices are preserved sub-parallel each other. They seem to have been a rigid paired structure, with distally symmetrical vanes composed of thick and probably rigid barbs. The morphology of this tail feather is not optimized for aerodynamical purposes, and based on the evidence at hand, it is probable that the mobility of the paired rectrices probably had some restrictions and may do not spread as in living birds. However, more evidence may be needed to support this statement.

The skeletal features and minute size sustain that UFRJ-DG 031 Av is a juvenile specimen^13^,^14^,^15^. In contrast, its plumage is very well developed, especially its elongate tail rectrices, which show well-differentiated and long vanes and scapus. In this aspect, the tail (and probably the entire body) plumage of these enantiornithines resembles the feathering of adult individuals of modern birds^15^,^39^. Notably, a well-developed plumage, especially ornamental rectricial feathers are also present in a young juvenile enantiornithine from Jehol^42^, indicating that these rectrices appeared early in their ontogeny^7^. If this interpretation is correct, it may indicate that there probably existed major differences between the development of the plumage in Enantiornithes and living birds, in which young individuals are devoid of long and well-differentiated tail rectrices^11^,^12^,^15^.

## Methods

### Phylogenetic analysis

Present phylogenetic analysis is based on the version of O'Connor and Zhou^31^ data set, which constitutes the most comprehensive analysis regarding the phylogeny of Enantiornithes. The matrix was only modified by the inclusion of UFRJ-DG 031 Av. The phylogenetic analysis was performed using TNT 1.1 (SI 3). All characters were equally weighted and treated as unordered. The strict consensus tree (SI) resulted on a large polytomy at the base of Enantiornithes that comprised most genera, including specimen UFRJ-DG 031 Av.

## Additional information

**How to cite this article:** de Souza Carvalho, I. *et al.* A mesozoic bird from Gondwana preserving feathers. *Nat. Commun.* 6:7141 doi: 10.1038/ncomms8141 (2015).

## Supplementary Material

Supplementary Information Supplementary Figures 1-7, Supplementary Notes 1-4 and Supplementary References
