# Supplementary material for: A Mesozoic bird from Gondwana preserving feathers
Source: Nat Commun. 2015 Jun 2;6:7141. doi: 10.1038/ncomms8141 (PMC4458891; doi:10.1038/ncomms8141)
Supplement: Supplementary Information — Supplementary Figures 1-7, Supplementary Notes 1-4 and Supplementary References [file ncomms8141-s1.pdf]

ESM 1. **Additional figures of UFRJ-DG 031 Av**

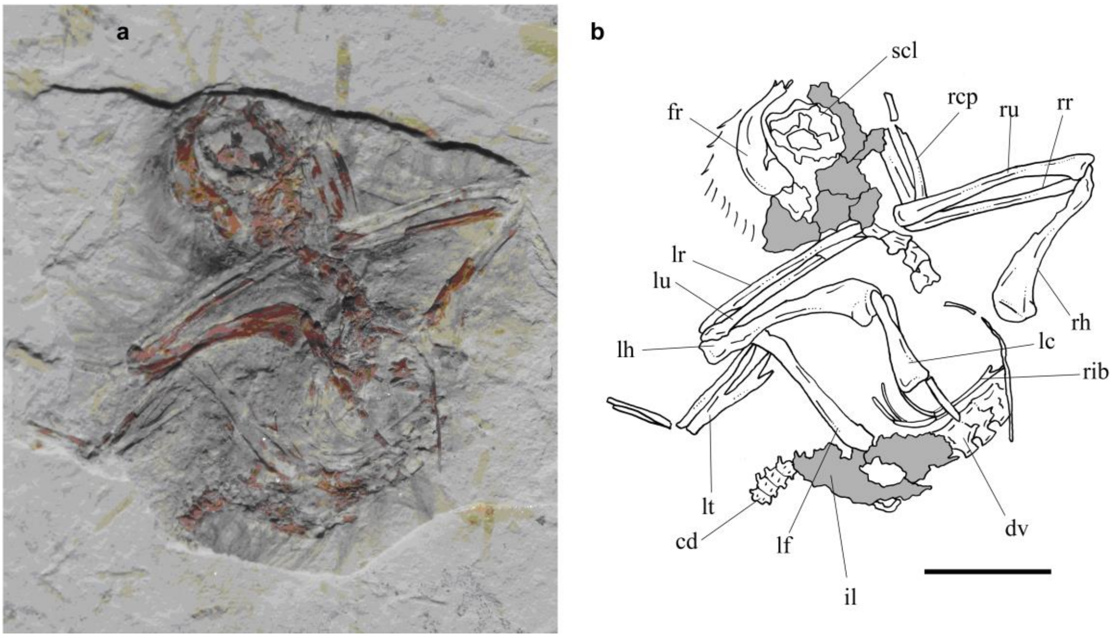

Supplementary Figure 1 Counterslab of UFRJ-DG 031 Av. **a**, main slab. **b**, interpretative drawing of the skeleton and feathers Abbreviations: cd, free caudal vertebrae; dv, dorsal vertebrae; fr, frontals; il, ilium; lc, left coracoid; lf, left femur; lh, left humerus; lr, left radius; lt, left tibiotarsus; lu, left ulna; rcp, right carpometacarpus; rh, right humerus; rib, dorsal rib; rr, right radius; ru, right ulna; scl, sclerotic ring. Scale bar, 1 cm.

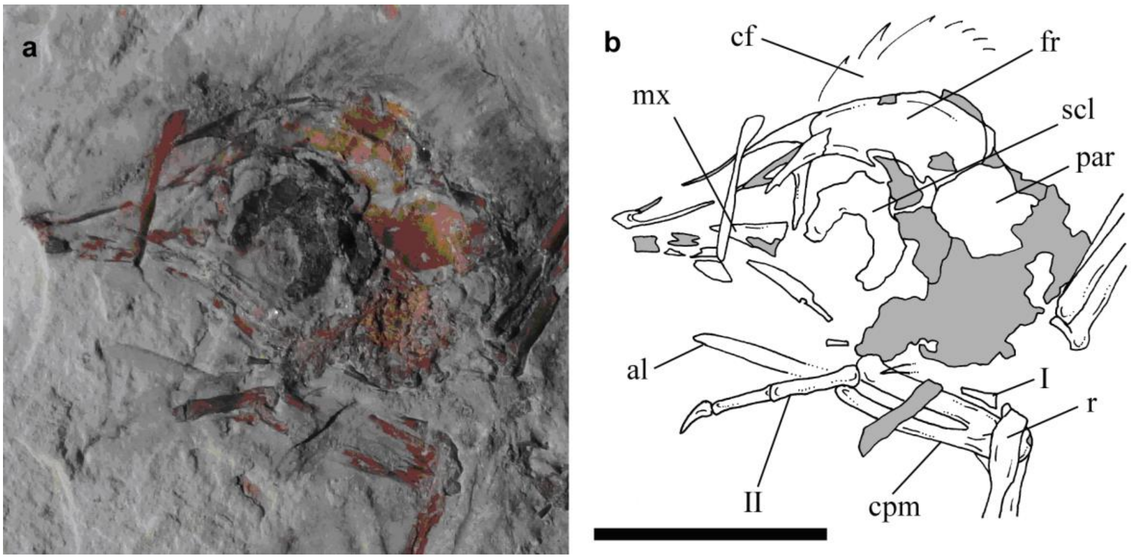

Supplementary Figure 2 ESM. **Details of cranial bones of UFRJ-DG 031 Av**. **a**, main slab. **b**, interpretative drawing. Abbreviations: al, alular feather; cf, cover feathers; cpm, carpometacarpus; fr, frontals; I, digit I; II, digit II; mx, maxilla; par, parietal; r, radius; scl, sclerotic ring. Scale bar, 1 cm.

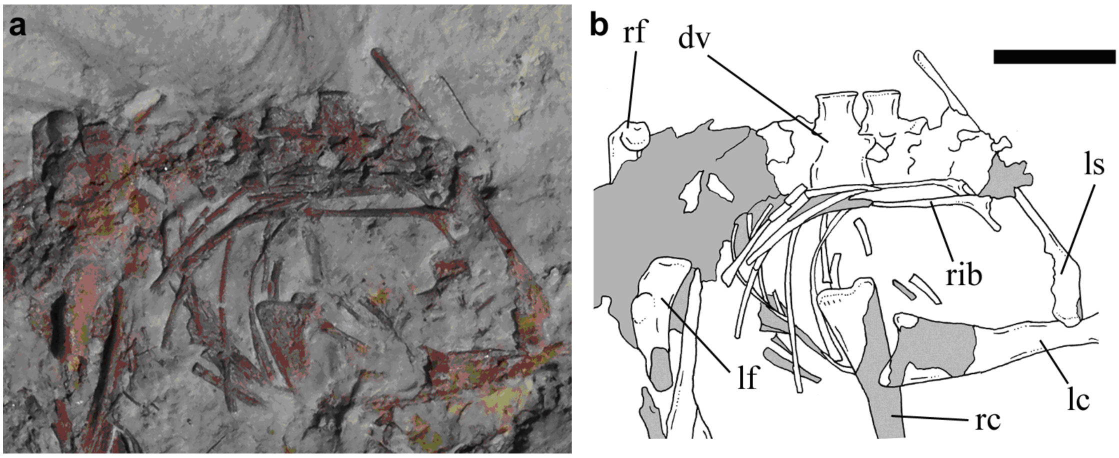

Supplementary Figure 3. **Detail of the thorax of UFRJ-DG 031 Av**. **a**, main slab. **b**, interpretative drawing. Abbreviations: dv, dorsal vertebrae; lc, left coracoid; lf, left femur; ls, left scapula; rc, right coracoid; rib, dorsal rib. Scale bar, 0,7 cm.

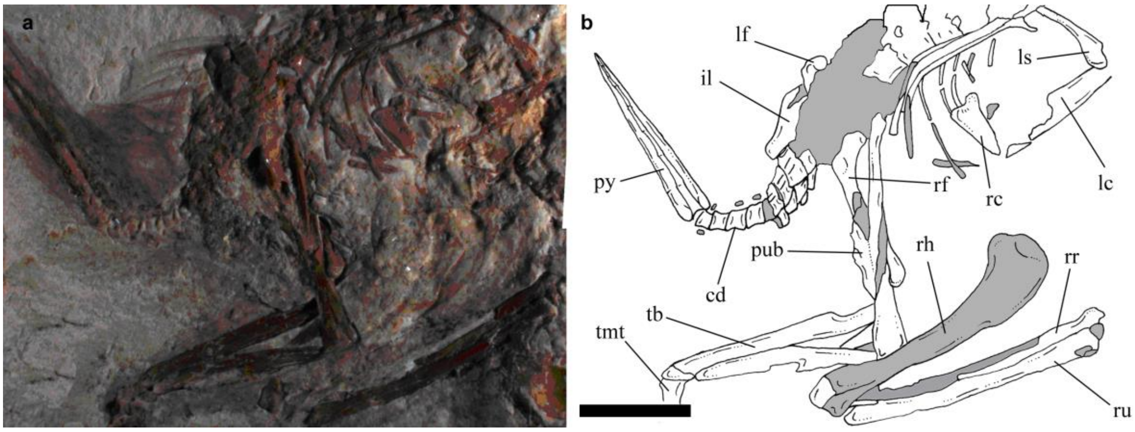

Supplementary Figure 4. **Details of the skeleton of UFRJ-DG 031 Av**. **a**, main slab. **b**, interpretative drawing. Abbreviations: cd, free caudal vertebrae; il, caudal end of left ilium; lc, left coracoid; lf, left femur; ls, left scapula; pub, pubes; py, pygostyle; rc, right coracoid; rh, right humerus; rr, right radius; ru, right ulna; tb, tibiotarsus; tmt, metatarsus. Scale bar, 0,2 cm.

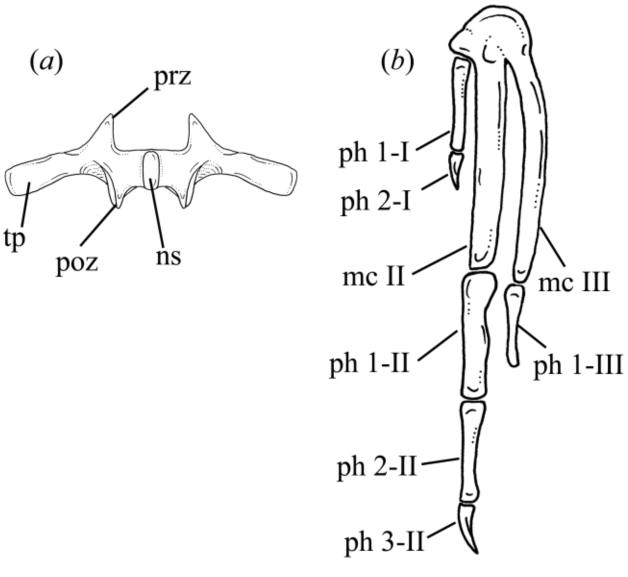

Supplementary Figure 5. **Reconstruction of selected elements of UFRJ-DG 031 Av**. **a**, anterior free caudal vertebra in dorsal view; **b**, right manus in lateral view.

Abbreviations: mc, metacarpal; ns, neural spine; ph, phalanx; poz, postzygapophyses; prz, prezygapophyses; tp, transverse process. Not to scale.

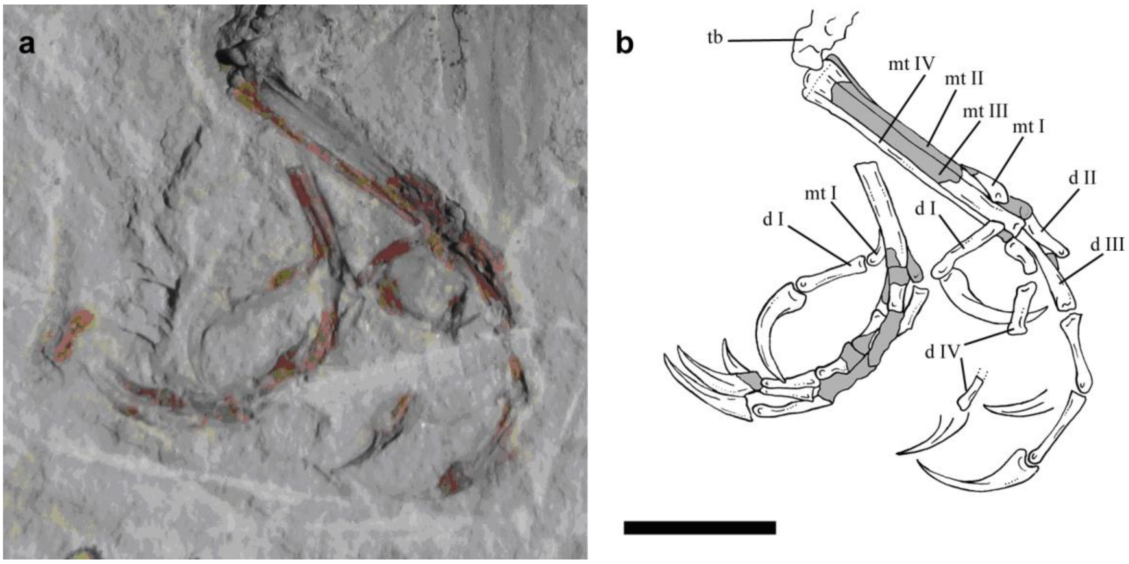

Supplementary Figure 6. **Details of the feet of UFRJ-DG 031 Av**. **a**, main slab. **b**, interpretative drawing. At left an incomplete left foot, at right a nearly complete right foot. Abbreviations: d, digit; I, one; II, two; III, three; mt, metatarsal; tb, tibiotarsus. Scale bar, 0,5 cm.

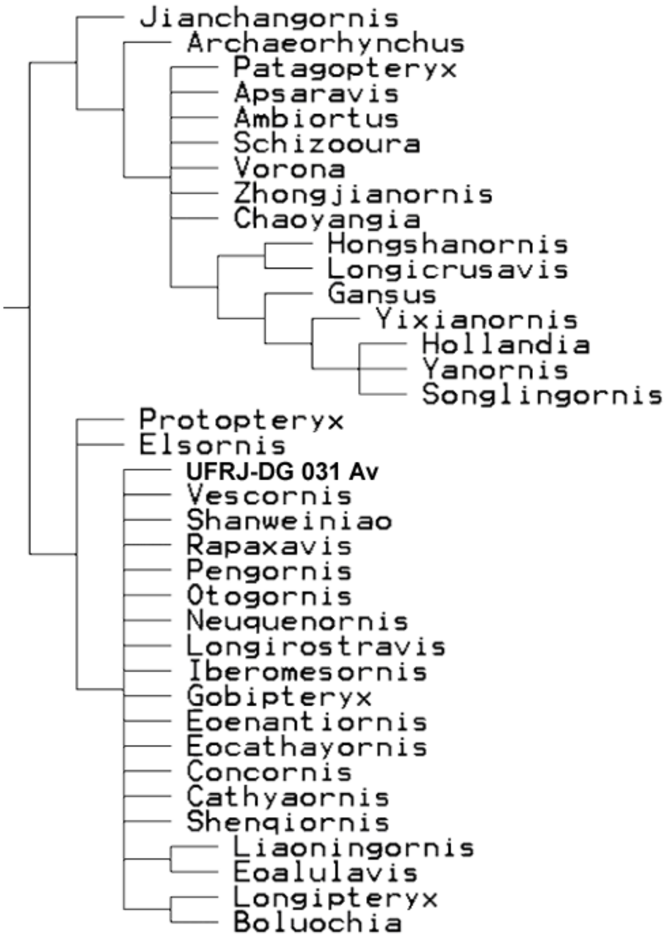

Supplementary Figure 7. Strict consensus tree showing the phylogenetic position of UFRJ-DG 031 Av within Enantiornithes.

Supplementary Note 1. Additional description of the skeleton of UFRJ-DG 031 Av.

UFRJ-DG 031 Av consists on a nearly complete skeleton, preserved in two slabs, including skull, nearly complete fore and hindlimbs, vertebral column and pectoral and pelvic girdles. However, as is usual in fossils preserved in two slabs, the bones are cracked. Some crushing and displacement of bones has occurred. Several bones, however, remain in articulation. The skull and neck are rotated ventrally with respect to the rest of the skeleton, and are exposed on the left side, whereas most of remaining skeleton is exposed on the right side. The skull and mandible suffered strong crushing and deformation, and most bones cannot be individualized. The pelvic girdle and hindlimb remain in anatomical position, whereas the pectoral girdle has been displaced ventrally with respect of the dorsal column. The minor slab shows better preserved bones than the main slab. It contains the impression of the skull, and both forelimbs and fragments of pectoral girdle. The ischia and sternum are not preserved.

The very small body size, large orbit, elongate caudal series, poorly developed proximal humerus and distal ends of other long bones (femur, tibia), as well as the lack of fusion in the metatarsus indicates that the specimen may be a juvenile

The skull is highly distorted and poorly preserved, and only some bones can be properly recognized. We identify several skull roof elements including parietals, frontals and nasal, as well as the maxilla and lacrimal bone. Some other elements located at the level of the snout, nearly perpendicular to the main axis of the skull are problematic, since they are much more robust compared to other cranial elements, and their morphology do not match with bones of the rostrum.

The parietals and frontals are dorsally convex, indicating a vaulted braincase. The snout is highly pointed and subtriangular in contour. The maxilla is subtriangular and

the presence of minute alveoli on its mid portion indicate the existence of small-sized teeth, which are not preserved in the slab. The lacrimal bone is incompletely preserved but shows a pillar-shaped ventral process. Due to weathering, remaining skull and dentary bones do not offer interesting anatomical information.

The vertebral column of UFRJ-DG 031 Av is represented by cervical, dorsal, sacral, and caudal vertebrae, but each element is very poorly preserved. Five cervical vertebrae have been preserved in articulation, and are exposed on their right side. The complete number of cervicals is unknown. The centra are rather elongate and lack any sign of lateral excavations or pleurocoels. A longitudinal ridge separates the centra from the neural arch. The neural spines are dorsoventrally tall and show a subrectangular contour in lateral view. Although incompletely preserved, vertebral centra are stouter and shorter than those of other enantiornithes, such as *Gobipteryx*. Six dorsal vertebrae are preserved. Although they are highly distorted, they show a conformation similar to that of other enantiornithes. The vertebral centra are relatively short, resembling the proportions of basal enantiornithes such as *Iberomesornis* [1], being different from derived enantiornithines such as *Gobipteryx*, in which they are longer [2] and resembles basal taxa, as for example, The centra exhibit a deep lateral longitudinal excavation, as frequently occurs in Enantiornithes [3]. The neural spine is well developed and subrectangular in contour, being slightly fan-shaped distally. Parapophyses are located high on the centrum, a diagnostic character of Enantiornithes [1].

Caudal vertebrae are poorly preserved, the is represented only by its transverse processes. There are 8 free caudal vertebrae. The second and third caudals are completely preserved, but exposed in dorsal view. The first half of the fourth caudal has been preserved as bone, whereas the posterior half has been preserved as a mold. Caudals fifth through eight are preserved as impressions of the ventral face of their

centra. The vertebral centra are not well-preserved, but appear exhibit biconvex articular surfaces. On caudals 2-4 the neural spine is transversely thick, and the transverse processes are very well-developed, robust, and craniocaudally expanded. A composite reconstruction of the neural arch of free caudals in dorsal view, indicates transversely compressed zygapophyses, thick neural spine, and a wide fossa lateral to the postzygapophyses, for the articulation of the prezygapophysis of the posterior vertebrae. This combination of characters is different from the more reduced caudal neural spine, transverse processes and zygapophyses seen in most enantiornithine taxa.

The pygostyle of UFRJ-DG 031 Av is a rod-like structure very similar to that present in other enantiornithes. It is composed by 8 caudal vertebrae, and is cone-shped, being longer than the combined length of free caudals. The proximal end is forked, showing two sub-parallel longitudinal processes, as is diagnostic of Enantiornithes [4]. Vertebral bodies are short and sub-cilindrical, and lack any sign of transverse processes. The pygostyle exhibits a longitudinally extended ventrolateral process, as frequently occurs among Enantiornithes [4]. The elongate tail feathers insert at the level of the third pygostyle vertebra.

No cervical ribs have been preserved, or they cannot be identified on the available cervicals. Most of the right dorsal ribs are preserved, as well as the distal tips of some left ribs. The rib pattern is similar to that described for the enantiornithine *Iberomesornis* [1]. The first dorsal rib is very long. Posterior dorsal ribs decrease in length. The proximal end of the ribs is bicapitate, and its distal end is slightly expanded for articulation with sternal ribs. There are some fragmentary preserved sternal ribs. These show a slightly expanded distal articular surface.

Both coracoids are preserved partially as natural molds, and are exposed in ventral view. They are elongate and narrow, strut-like bones. The proximal end is typical of

enantiornithine birds, showing a highly reduced procoracoid and a convex boss for articulation with the scapula. The acrocoracoid is reduced and rounded in contour. The lateral margin of coracoid is convex, whereas the medial one is slightly concave. As in most Enantiornithes the lateral process is absent [2]. The distal margin of the bone appears to be only slightly concave.

The left scapula is preserved in dorsal view. The proximal end is badly damaged, and most structures are obscure. The acromion is well-developed and subtriangular in contour, and is laterally oriented, a condition similar to that of other Enantiornithes. The distal end of the scapular blade is acute and transversely compressed.

Both humeri are exposed in posterior view. The right humerus is nearly complete, whereas the left one is eroded on its distal half. The humeral head is rounded in contrast with the majority of Enantiornithes, in which it is apomorphycally saddle-shaped [1]. This head is located central to the main axis of the bone, as commonly occurs in enantiornithine birds (e.g., *Iberomesornis*, *Gobipteryx*; [2,5]). On its centre exhibits a poorly concave longitudinal groove. The bicipital tubercle is a poorly differentiated and rounded process, the capital groove and the transverse ligamental groove are absent, a combination of characters that contrast with more derived Enantiornithes (e.g., *Martinavis, Enantiornis, Gobipteryx, Halimornis* [6]). The bicipital crest is absent, a plesiomorphic condition present in Enantiornithes and *Archaeopteryx* [2]. The distal end of the humerus is transversely expanded, although not at the degree seen in Euenantiornithes. There is not clear evidence of the presence of a distal caudal fossa. The distal condyles are not exposed; however, in caudal view, a ventral extension of the distal margin indicates that the dorsal condyle was nearly sub-parallel to the humeral diaphysis, a diagnostic condition to Enantiornithes [1].

The left and right ulnae are preserved in posterior aspect. Both are highly fragmented and show poorly preserved proximal and distal ends, precluding the description of most features. The ulna is nearly as long as the humerus, a condition that contrast with that of derived Enantiornithes, including *Elbretornis* and *Enantiornis*, in which the ulna is much shorter than humerus [6]. The proximal end of the bone shows a well-developed olecranon and the external condyle is prominent and rounded. The distal end of ulna is nearly flat and subcuadrangular in contour, contrasting with the pulley-shaped morphology seen in other Enantiornithes [6].

Left and right radii are preserved in posterior view. The proximal end shows a well developed median concavity. In posterior view its distal end is rounded in contour. It shows a longitudinal groove, as diagnostic of Enantiornithes [3]. The shaft of the radius appears to the wider proximally than its distal end, a peculiar condition shared with *Iberomesornis* and *Enantiornis* [1].

UFRJ-DG 031 Av manus is sub-equal to total ulnar length. The left carpometacarpus has been preserved in lateral view, whereas the right carpometacarpus is represented by highly crushed scraps of bone. Proximally, the carpometacarpus is tightly-fused, whereas the distal end is unfused. The proximal end of carpometacarpus is badly crushed, with por anatomical details. A small fragment of bone may represent metacarpal I. Phalanx 1-I is elongate, although its incompleteness precludes estimation of its total length. The distal end of ungual 2-I is preserved; it is acute and slightly curved. Metacarpal II is shorter and thinner than metacarpal III, a diagnostic condition of Euenantiornithes [6]. Phalanx 1-II is robust and subrectangular in contour, lacking of mid-constriction. Phalanx 2-II is preserved mainly as a bone impression. It is thinner and slightly shorter than 1-II and shows a well-defined mid constriction. Ungual 3-II is acute and sligtly curved, and appears to be sub-equal in size and morphology to ungual

2-I. Only the base of phalanx 1-III has been preserved, indicating a reduced digit III, as frequently occurs in birds.

The right ilium is represented by part of the acetabulum. The acetabular portion of ilium lacks of supracetabular crest, and the presence of a caudal antitrochanter can be observed on the posterior margin of the acetabulum. The left ilium preserves its posterior blade. It is subtriangular in contour and dorsoventrally low.

The pubes are observed in caudal view but their proximal and distal ends are broken. A well-developed pubic symphysis appears to be present.

The right femur is exposed in caudal view; its mid-shaft has been broken away, and its distal end is badly damaged. The left femur is hidden by a large amount of scraps of indeterminate bones, the right femur and the pubes. The only preserved portions are its proximal end in medial view, and the medial condyle in posterior view. The length of the left femur does not matches with the right one (see measurements), suggesting that there was some movement of proximal and distal parts. The femoral shaft appears to be nearly straight. The femoral head is anterodorsally oriented, and a fovea capitis appears to be absent. It shows a continuous articular surface with the trochanteric crest. The latter one is well developed, and proximally reached the level of the proximal margin of the femoral head. The iliotrochanteric drepression is wide and deep, subtriangular in contour. In caudal view, a bulbous posterior trochanter is present distal to the trochanteric crest. The distal end is not very well preserved. A patellar groove appears to be absent, and the medial condyle is bulbous and ellipsoidal in side view.

Both tibiotarsi show a badly crushed surface, and their distal and proximal ends are poorly preserved. The right tibiotarsus is exposed in lateral view, whereas the left tibiotarsus is exposed in medial view. The left tibiotarsus shows hidden proximal and

distal ends. The tibiotarsus is much shorter than the femur, and is subequal in length to the metatarsals. Due to its poor preservation additional data cannot be afforded.

The proximalmost portion of the metatarsals is not fused, and a small metatarsal cap is absent. In posterior view the hypotarsus is absent. The right metatarsals are exposed in posterior aspect, most of the shaft of metatarsals III and II are preserved as impressions. The left metatarsals are exposed in anterior view, but their proximal halves are hidden by the right metatarsals. The metatarsals are elongate and transversely narrow. Metatarsals are unfused along most of its length. Metatarsal IV is transversely compressed, specially at its distal end, and is thinner than other metatarsals, as diagnostic of Enantiornithes [1]. Its distal end is very transversely compressed and shows a ginglymoid articulation. Metatarsal I is small and has strongly posteriorly deflected distal condyles, and distal condyles are proximally joined, both characters considered as diagnostic of Enantiornithes [1]. Metatarsal II is relatively robust. Its distal end and the articular surface of phalanx 1-II does not appears to be wider than metatarsal III, in contrast with remaining Enantiornithes [1]. Metatarsal III is the stouter bone of the foot, it is transversely wide and homogeneous in width along all its length. Its distal ginglymoid is poorly preserved.

Pedal phalanges are relatively well preserved, although most unguals are preserved only as impressions. Non-ungual phalanges 1-IV and 2-IV are relatively elongate and its combined length clearly surpasses the distal end of phalanx 1-III, whereas in *Iberomesornis* they do not surpasses such level [1]. Phalanges 3-IV and 4-IV are not preserved. Non-ungual phalanges of pedal digit III are very narrow and extremelly elongate, being much longer than metatarsal III. In other Enantiornithes (e.g., *Iberomesornis* [1]) the combined length of these phalanges is subequal or shorter than metatarsal III length. Phalanx 1-III is the stouter of the foot and shows a very wide and

transversely expanded proximal end. Phalanx 2-IIII is not well preserved; it was relatively slender and constitutes the shortest non-ungual phalanx of the foot. Phalanx 3-III is very elongate and narrow, being the more gracile element of the pes. Its distal ginglymoid appears to be small and only slightly excavated. Phalanges 1-II and 2-II are elongate and subequal in length and morphology each other. Phalanx 1-I is very elongate and robust, being the stouter element of the foot, as commonly occurs in Enantiornithes [7]. Pedal unguals are elongate, and show a slightly curved ventral surface. Ungual 5-IV does not preserves its proximal half, but available information suggests that it was relatively elongate and smaller than other unguals. Ungual 4-III is relatively robust and is larger than unguals of digit II and III. Ungual 3-II is subequal in length and morphology to other unguals of the foot. Pedal ungual of digit I with very curved ventral margin, much more than remaining unguals.

Remarkably, the specimen preserves different kind of feathers around the skeleton. The skeleton of UFRJ-DG 031 Av is covered by filamentous feathers, including a crown of feathers located at the top of the head. 10 secondary asymmetrical remiges anchored on the forearm are preserved. None of the remiges are completely preserved, so its total length and morphology are nearly unknown. Left and right alulae are represented by feathers attached to digit I, a condition widespread among Enantiornithes [8]. At least three alular feathers have been preserved on the left hand. Alular feathers are asymmetrical, a condition widespread among birds.

A preserved pair os tail feathers is rather elongate, being 30% longer than total body length. They insert on the third pygostylian vertebra and conform the typical ribbon-like morphology present in Enantiornithes and Confusiusornithidae [9] these tail feathers gradually increase in width distally. The calamus of UFRJ-DG 031 Av is extremelly well-developed, and shows on its midline a narrow longitudinal groove that runs from

the base to the distal end of the feather. A cross-section at the base of the calamus indicates that the structure was nearly flat, whereas the cross-section at the mid-length indicates that at this portion the calamus was very convex. A reconstruction indicates that mid calamus was nearly 8-shaped in cross section. At the distal third, the calamus gradually flattens towards the rachis. The first sign of barbs is observed at the distal half of the tail, and barb size increases towards its distal end. The barbs are clearly distinguishable at the tip of the tail (approximately the distal 2 centimeters of the tail feather). Each barb was dorsoventrally thick and dorsally convex, and of uniform thickness and width for most of its length. They are angled at approximate 16º from the rachis and lack any sign of interlocking barbules. Both vanes are sub-equal, suggesting a symmetrical feather. At the distal end, and near its contact with the third pygostylian vertebra, the calamus narrows abruptly. One remarkable aspect of these ribbon-like feathers is the existence at the base of each feather of a longitudinal row of five brown spots at the calamus, probably representing the original tail coloration of UFRJ-DG 031 Av. No remains of hindlimb feathers are recognized.

Supplementary Note 2. Measurements of UFRJ-DG 031 Av.

Right humerus, total length: 14 mm.

Coracoid, total length: 7.7 mm

Left ulna, total length: 13.3 mm

Left metacarpal III, total length: 7.2 mm.

Left manual phalanx 1-III, total length: 3.2 mm

Right femur, total length*: 11.7 mm

Left femur, total length: 12.8 mm

Left tibia, total length*: 12 mm

Left metatarsal III, total length: 8.9 mm

Right pedal phalanx 1-I, total length: 2.6 mm

Right pedal ungual of digit I, total length: 3.5 mm

Pygostyle, total length: 9.4 mm

Caudal series, total length: 8.3 mm

Caudal tail feather, total length: 79.9 mm

Preserved portion of right remiges, total length of longest feather: 10.3 mm

Preserved portion of left remiges, total length of longest feather: 8.6 mm

* Indicates estimated size due to incomplete preservation of the element.

Supplementary Note 3. Phylogenetic analysis

Present phylogenetic analysis is based on the version of O´Connor and Zhou [10] data set, which constitutes the most comprehensive analysis regarding the phylogeny of Enantiornithes. The matrix was only modified by the inclusion of UFRJ-DG 031 Av. The data matrix is composed of 245 characters distributed among 51 taxa.

The phylogenetic analysis was performed using TNT 1.1 [11]. All characters were equally weighted and treated as unordered. Heuristic searches were performed after 1,000 pseudoreplications of WAG+TBR search strategy, with 10 random addition sequences after each search and 100 trees were saved at each replicate. The phylogenetic analysis resulted in the recovery of 82 Most Parsimonious Trees (MPTs), of 71 steps, with a consistency index of 0.40, and a retention index of 0.71. The strict consensus tree (Supplementary Figure 7) resulted on a large polytomy at the base of Enantiornithes that comprised most genera, including UFRJ-DG 031 Av.

Supplementary Note 4. Scoring for UFRJ-DG 031 Av in the data matrix of O´Connor and Zhou [10]

UFRJ-DG 031 Av

??????1??0??????00????0?????24
010?0110000111110100?1??000?0?????????????????????010?0100002?1???????
???11?????1???1???0?0100001200100?122??????????????????01?0012????????
??????????100?0010??01??20?0?0?1210

Supplementary references

1- Sereno, P.C. *Iberomesornis romerali* (Ornithothoraces, Aves) re-evaluated as an enantiornithine bird *Neues Jahr. Geol. Paläont. Abhand.* **215**, 365–395 (2000).

2- Kurochkin, E.N. A new enantiornithid of the Mongolian Late Cretaceous, and a general appraisal of the Infraclass Enantiornithes (Aves). *Russ. Acad. Sci., Special Issue*: 1–50 (1996).

3- Chiappe, L.M. & Calvo, J.O. *Neuquenornis volans,* a new Late Cretaceous bird (Enantiornithes: Avisauridae) from Patagonia, Argentina. *J. Vert. Paleont.* **14**, 230–246 (1994).

4- Chuong, C.M., Wu, P., Zhang, F.C., Xu, X., Yu, M., Widelitz, R.B., Jiang, T.X. & Hou, L. Adaptation to the sky: Defining the feather with integument fossils from Mesozoic China and experimental evidence from molecular laboratories. *J. Exp. Zool. B Mol. Dev. Evol.* ***298***, 42–56 (2003).

5- Sanz, J.L. & Bonaparte, J.F. A New Order of Birds (Class Aves) from the Lower Cretaceous of Spain. Nat. Hist. Mus. of Los Angeles County Contrib. Sci. 36, 38–49 (1992).

6- Chiappe, L.M. & Walker, A. Skeletal morphology and systematics of the Cretaceous Euenantiornithes (Ornithothoraces: Enantiornithes). In *Mesozoic birds: above the heads of dinosaurs* (eds. Chiappe LM, Witmer L), pp. 240–267. Univ. California press (2002).

7- Martin, L.D. The Enantiornithes: terrestrial birds of the Cretaceous. *Cour. Forsch. Senck.* **181**, 23–36 (1995).

8- Sanz, J.L., Chiappe, L.M., Pérez–Moreno, B.P., Buscalioni, A.D., Moratalla, J.J., Ortega, F. & Poyato–Ariza, F.J. An Early Cretaceous bird from Spain and its implications for the evolution of avian flight. *Nature* **382**, 442–445 (1996).

9- O'Connor, J.K., Chiappe, L.M., Chuong, C., Bottjer, D.J. & You, H. Homology and Potential Cellular and Molecular Mechanisms for the Development of Unique Feather Morphologies in Early Birds. *Geosciences* **2**, 157–177 (2012).

10- O'Connor, J.K. & Zhou, Z. A redescription of *Chaoyangia beishanensis* (Aves) and a comprehensive phylogeny of Mesozoic birds. *J. Syst. Paleont.* **11**, 889-906 (2012).

11- Goloboff PJ, Farris J, Nixon K. 2008. A free program for phylogenetic analysis. *Cladistics* **24**: 774-786.
